# Advancing clinical evaluation programs in nursing education in Saudi Arabia: a cross-sectional study of students’ and instructors’ perspectives

**DOI:** 10.3389/fmed.2026.1715157

**Published:** 2026-02-09

**Authors:** Mohammad Dagamseh, Suhair Al-Ghabeesh, Abdulai Abukari

**Affiliations:** 1 Nursing Department, Arabian Gulf University, Manama, Bahrain; 2 Nursing Department, Al-Zaytoonah University of Jordan, Amman, Jordan; 3 The British University in Dubai, Dubai, United Arab Emirates

**Keywords:** clinical evaluation, clinical instructors, competency based assessment, nursing education, Saudi Arabia

## Abstract

**Background:**

Clinical Evaluation Programs (CEPs) are a fundamental component of nursing education because they facilitate the structured assessment of students’ clinical competence in conjunction with theoretical instruction. The effectiveness of these programs is dependent on clarity, consistency, and acceptability by stakeholders.

**Aim:**

This study aimed to examine the perceptions of nursing students and clinical instructors regarding CEPs in Riyadh, Saudi Arabia, along with the demographic factors associated with these perceptions.

**Methods:**

A quantitative cross-sectional study was conducted involving 303 undergraduate nursing students and 61 clinical instructors. The data were collected using a validated questionnaire. Mann–Whitney U and Kruskal–Wallis tests were performed to examine group differences, followed by regression analysis to assess the associations between demographic variables and CEP perception scores.

**Results:**

Overall, perceptions of CEPs were moderate, with mean scores close to the midpoint of the 5-point Likert scale for both students (*M* = 3.26) and instructors (*M* = 3.50). There were significant associations between CEP perceptions and selected demographic variables, including age, academic level, gender, and marital status, with small to moderate effect size (ES).

**Conclusion:**

Although CEPs are regarded as essential elements of clinical education, improvements in clarity, feedback processes, and standardization are required to enhance their effectiveness and educational impact.

## Introduction

Nursing has evolved from a task-oriented care role into a complex, evidence-based healthcare field that demands advanced clinical judgment, skill, and professional competence. It has been reported that contemporary nursing education requires a robust and reliable framework to ensure that graduates are adequately prepared to meet the complex demands of modern healthcare practice (1). Within this context, Clinical Evaluation Programs (CEPs) have emerged as a fundamental component of nursing education, providing structured mechanisms for assessing clinical competence and bridging the gap between theory and practice (1).

Nursing education is dual in nature, requiring students to acquire theoretical knowledge in the classroom learning while also developing practical skills in the clinic. Clinical placements allow students to apply what they have learned, gain proficiency in specific skills, and enhance their decision-making abilities in real-life situations (2). Additionally, CEPs ensure a comprehensive assessment of technical, cognitive, and interpersonal skills of students. This comprehensive approach helps confirm that students are competent in providing quality care to patients (3). However, a significant challenge is achieving consistency and fairness in clinical assessments.

To address this challenge, CEPs use a variety of assessment strategies, including direct clinical observation, competency-based assessments, and Objective Structured Clinical Examinations (OSCEs). OSCEs, in particular, are widely recognized for their structured format, which involves simulated patients, and their ability to objectively assess clinical skills (4). Moreover, other workplace-based assessment methods, such as Direct Observation of Procedural Skills (DOPS), Mini Clinical Evaluation Exercise (Mini-CEX), and Clinical Work Sampling (CWS), have been developed to provide immediate feedback in clinical settings, thereby enhancing reflective learning (5, 6). Despite their widespread adoption, these methods are frequently criticized for inconsistency, subjectivity, and potential assessor bias (7).

Clinical assessment is a continuous, practice-based activity that aims to identify knowledge gaps, improve clinical skills, and foster professional behavior (8). In Saudi Arabia, initiatives aligned with Vision 2030 have emphasized the use of structured CEPs and advanced assessment approaches, including simulation-based learning, web-based assessment platforms, and standardized evaluation tools, to enhance the quality of clinical education. Nevertheless, the implementation of these tools remains inconsistent across institutions (9, 10).

The role of clinical instructors (CIs) in supervising students, providing formative feedback, and making summative judgments regarding clinical competence is an important factor in determining the efficacy of CEPs. Effective clinical instructors require strong professional expertise, instructional competency, communication skills, and professionalism, all of which have a significant influence on students’ learning experiences. However, clinical instructors often face competing demands related to workload and clinical responsibilities and may receive limited formal training in assessment methodologies. These challenges can lead to discrepancies between instructors’ perceived competence, students’ learning experiences, and institutional expectations (11).

From a student’s perspective, CEPs play a crucial role in shaping learning outcomes, boosting self-confidence, and enhancing satisfaction with clinical learning experiences. Supportive supervision, transparent evaluation criteria, and constructive feedback are key elements that promote positive clinical learning environments and facilitate the application of theory to practice (12, 13). In contrast, inadequate guidance, unclear expectations, clinical stressors, and poorly structured assessments may negatively affect skill acquisition and the overall learning experience (14). As a result, both students’ and faculty members’ perceptions of CEPs are influenced by multiple contextual and individual factors.

Despite the growing body of literature on clinical evaluation methods and instruments, a significant research gap remains regarding the simultaneous examination of nursing students’ and clinical instructors’ perceptions regarding CEPs within the Saudi Arabian context. Existing studies have often explored evaluation tools or stakeholder roles in isolation, providing limited insights into areas of alignment or divergence between students’ and instructors’ perspectives. Addressing this gap is essential to enhancing CEP effectiveness, reducing subjectivity, and improving the quality of nursing education in alignment with Saudi Vision 2030.

Accordingly, the present study aims to evaluate the effectiveness and perceived impact of Clinical Evaluation Programs in nursing education by examining and comparing the perceptions of nursing students and clinical instructors across Saudi nursing colleges. Specifically, the study explores how demographic factors influence these perceptions, thereby providing evidence-based insights to inform the improvement of CEP implementation and quality assurance practices.

## Methods

### Design

A cross-sectional quantitative design was used to explore the perceptions of nursing students and clinical instructors regarding Clinical Evaluation Programs, as well as the influence of demographic factors on these perceptions.

### Setting

The study was conducted at two government nursing colleges in Riyadh, Saudi Arabia. College “A” is exclusively for female students, whereas College “B” is a large government college that enrolls male and female students in separate educational tracks. Nursing education in Saudi Arabia follows a 5-year Bachelor of Nursing program, which consists of approximately 70% theoretical coursework and 30% clinical practice; however, these proportions may vary slightly depending on the specific requirements of each institution.

Formal clinical education becomes more intensive after level 4, with students in levels 5–8 participating in clinical placements 2–3 days a week. During this phase, students are monitored and assessed by qualified clinical instructors holding bachelor’s and/or master’s degrees in nursing or related healthcare disciplines. Student progress is evaluated using competency-based checklists, nursing care plans, direct observation, and simulated clinical scenarios in accordance with institutional CEPs (9, 15).

### Population and sampling

#### Target and accessible populations

The target population consisted of all undergraduate nursing students enrolled in Bachelor of Nursing programs in Saudi Arabia who participated in CEPs and clinical instructors who evaluated these students.

During the data collection period, the accessible population included nursing students enrolled in levels 5–8 and clinical instructors supervising these levels at two selected governmental nursing colleges in Riyadh. The total accessible population during the 2016/2017 academic year consisted of approximately 1,600 nursing students and 90 clinical instructors. The instructor-to-student ratio ranged from 1:15 to 1:25, based on institutional policy and clinical module requirements.

##### Inclusion and exclusion criteria

Participants were eligible for inclusion if they met the following criteria:

1. Nursing students at level 5 and above in the Bachelor of Nursing Program. Students below level 5 were excluded because levels 5–8 represent the formal phase of advanced clinical education in Saudi nursing programs, during which structured CEPs are consistently implemented. At level 4, clinical exposure is primarily observational, with limited responsibility for independent clinical performance and minimal use of formal competency-based evaluation.
2. Clinical instructors responsible for supervising and evaluating students at levels 5–8.
3. Direct experience with CEPs.
4. Ability to read and understand English.
5. Willingness to provide informed consent.

##### Sampling technique and rationale

Convenience sampling was employed to recruit both nursing students and clinical instructors. This approach was considered appropriate due to logistical limitations, restricted institutional access, and the voluntary nature of participation across gender-segregated academic tracks. While stratified or quota sampling could have improved representativeness, institutional scheduling constraints, gender-segregated campuses, and variable clinical rotations limited the feasibility of proportional stratification. These limitations are acknowledged as factors affecting the generalizability of the findings.

### Sample size calculation

Sample size estimation was conducted using G* Power software (version 3.1). For nursing students, calculations were based on an independent samples *t*-test with a medium effect size (*d* = 0.5), an alpha level of 0.05, and a power of 0.80, yielding a minimum required sample size of 267. To account for potential non-responses, an additional 10% was added, resulting in a target sample size of 303 students.

For clinical instructors, the same statistical assumptions were applied, resulting in a minimum required sample size of 61 instructors. Data collection continued until the required sample sizes for both groups were achieved.

### Participant recruitment procedures

Following ethical approval, participant recruitment was conducted over 3 months (December 2022 to March 2023). Access to research settings was facilitated through coordination with college administrations and clinical education units. Invitations to participate were distributed via institutional email and WhatsApp.

The invitation outlined the study purpose, inclusion criteria, voluntary nature of participation, and confidentiality assurances. Participants accessed the online survey at their convenience. Reminder messages were sent periodically until the target sample size was reached, after which the survey link was deactivated.

### Research instrument

Data were gathered using an adapted structured self-administered questionnaire, referred to as “a revised clinical evaluation tool,” developed by Krautscheid et al. (16). Krautscheid et al. previously validated this instrument for use in relation to perceptions of clinical evaluation and instructional efficacy in nursing education, and previous validation research has shown strong psychometric qualities, with alpha levels greater than 0.90.

Although the original questionnaire was developed in English, minor wording modifications were made to enhance contextual relevance and clarity within the Saudi nursing education setting. These changes involved substituting locally used academic and clinical terms (e.g., replacing “clinical faculty” with “clinical instructor” and “course outcomes” with “clinical learning objectives”) without altering the meaning of the items or the structure of the constructs. Permission to adapt the instrument for research purposes was obtained from the original developer prior to data collection.

The last questionnaire was divided into three parts:

1. Demographic variables, such as designation (student or instructor), age, gender, academic level or years of experience, and institution.
2. Twelve items on a Likert scale, consisting of CEP evaluation (structure, relevance, and application) and Clinical Evaluation Process (fairness, clarity, and effectiveness).
3. An optional section for additional comments and contact information for follow-up.

Item analysis was conducted using the 5-point Likert scale that ranges from “strongly disagree” to “strongly agree.”

### Reliability and validity

Cronbach’s alpha was used to test internal consistency reliability, and coefficients of 0.955 for students and 0.947 for clinical instructors were found to be excellent. Content validity was verified through expert review, and prior international use of the instrument supports its construct validity. Nonetheless, relying on self-reported data introduces potential respondent bias.

### Data management and statistical analysis

Survey data were exported from the online platform into statistical analysis software. Surveys with more than 10% missing data were excluded, while minor missing data were addressed using appropriate deletion methods. Descriptive and inferential statistics were used to address the study objectives.

### Ethical considerations

Ethical approval was obtained from the Institutional Review Boards (IRB) of the British University in Dubai, King Saud University, and Princess Nourah bint Abdulrahman University (Approval date: 22/08/2022; Approval No. E-22-7225 and HAP-01-R-059). The researcher also completed the NCBE Research Bioethics Course in June 2022.

Participant anonymity was ensured by collecting no identifying information and reporting data in aggregate form. Institutional identities were anonymized. Participants were informed that participation or non-participation would not affect academic grades or professional evaluations. Electronic informed consent was obtained before accessing the survey.

## Results

Data analysis was conducted alongside data collection to ensure findings were aligned with the research questions. The cross-sectional survey examined nursing students (level 5 or above) and clinical instructors. Research Question 1 explored perceptions of Clinical Evaluation Programs (CEPs) by using SPSS for descriptive analysis, which helped identify trends and areas that require improvement.

Research Question 2 analyzed the relationship between perceptions and demographics such as age, gender, designation, and years of study. Significant correlations showed how these factors influenced perceptions of CEP.

Descriptive statistics summarized demographics and perceptions, while Mann–Whitney U and Kruskal–Wallis tests assessed differences across groups, using a significance threshold set at *p* < 0.05.

The Shapiro–Wilk test confirmed non-normality for both groups (students *W* = 0.875–0.945; instructors *W* = 0.890–0.935; *p* < 0.001). This finding supported the use of non-parametric methods, ensuring a valid and reliable analysis of CEP perceptions.

### Demographic characteristics of study participants

The demographic characteristics of nursing students (*n* = 303) and CIs (*n* = 61) are summarized in Table 1. The majority of the students were aged 21–23 (52%), followed by those aged 18–20 (26%), with smaller proportions in older age groups. Female students represented 60% of the participants, aligning with common trends in nursing education. Additionally, the majority of participants were single (92%). Students were evenly distributed across levels, with 20% in level 5, 24% in level 6, 25% in level 7, and 31% in level 8. University affiliation showed that 61% were from College A and 39% were from College B.

**Table 1:** Students’ and CI’s demographic characteristics (*n* = 364).

Variable		Frequency	%
Student’s age group	18–20	80	26
	21–23	158	52
	24–26	39	13
	27–29	24	8
	>29	2	1
CI’s age group	25–30	2	3
	31–35	24	39
	36–40	26	43
	41–45	7	12
	>45	2	3
Student’s gender	Male	183	60
	Female	120	30
CI’s gender	Male	28	46
	Female	33	54
Student’s marital status	Married	23	8
	Single	280	92
CI’s marital status	Single	13	21
	Married	48	79
Student’s level	5.00	62	20
	6.00	73	24
	7.00	75	25
	8.00	93	31
CI’s level of instruction	5.00	5	8
	6.00	14	23
	7.00	23	38
	8.00	19	31
Student’s University of Study	A	186	61
	B	117	39
CI’s University of teaching	A	35	57
	B	26	43
CI’s variable		Median	IQR
Years of experience		14	15
Monthly income		15,227	5,220
Teaching Hours/week		22	13

The majority of CIs were aged 36–40 (43%), with only 3% above the age of 45 years, reflecting a mid-career workforce. Female CIs represented 54% and male CIs represented 46%, with 79% being married. Instruction was concentrated at advanced levels, with 38% teaching level 7 and 31% teaching level 8. College A accounted for 57% of the participants, while College B accounted for 43%. CIs had a median of 14 years of experience, a monthly income of 15,227 SAR, and taught a median of 22 h per week, highlighting their professional experience and workload.

### Students’ perceptions of the clinical evaluation program

Table 2 summarizes the descriptive statistics of students’ and instructors’ perceptions of the CEP across various dimensions. The responses showed mixed views about its effectiveness in documenting progress and meeting objectives. Approximately 38% of students disagreed or were neutral about its role in tracking outcomes, and 40% felt similarly about its alignment with course objectives.

**Table 2:** Students’ and clinical instructors’ perceptions of clinical evaluation programs (CEPs).

CEP dimension	Students mean (SD)	Students median (IQR)	Instructors mean (SD)	Instructors median (IQR)
Documentation of clinical progress	3.22 (0.81)	3.00 (2.00)	2.91 (0.87)	3.00 (2.10)
Alignment with course objectives	3.20 (0.84)	3.00 (2.00)	2.88 (0.89)	3.00 (2.00)
Patient safety documentation	3.18 (0.86)	3.00 (2.00)	2.75 (0.92)	3.00 (2.00)
Identification of areas for improvement	3.27 (0.79)	3.00 (2.00)	2.83 (0.88)	3.00 (2.00)
Timeliness of feedback	3.14 (0.88)	3.00 (2.00)	2.69 (0.91)	2.50 (2.00)
Clarity of evaluation criteria	3.21 (0.82)	3.00 (2.00)	2.74 (0.90)	2.50 (2.00)
Usability of evaluation tools	3.25 (0.80)	3.00 (2.00)	2.78 (0.86)	3.00 (2.00)
Adequacy of time allocation	3.19 (0.85)	3.00 (2.00)	2.61 (0.94)	2.00 (2.00)
Preparation/orientation for CEP	3.16 (0.83)	3.00 (2.00)	2.67 (0.90)	2.00 (2.00)
Total CEP score	3.00 (0.77)	3.00 (2.05)	2.76 (0.81)	2.76 (2.09)

Higher scores indicate more positive perceptions. CEP, Clinical Evaluation Program.

Regarding safety documentation, 43% expressed concerns, indicating a need for improvement. While 39% agreed that the program identified areas that require improvement, 40% disagreed or were neutral. Similarly, 43% were dissatisfied or neutral about timely opportunities for improvement.

The clarity of CEP instructions and criteria received mixed feedback: 43% of respondents found them clear, while an equal proportion disagreed or were neutral. The usability of CEP tools was rated favorably by 44%, but 40% identified areas that need improvement.

Time allocation was appropriate for 44%; however, 38% had concerns. While 37% felt prepared for participation, a similar percentage indicated dissatisfaction.

Overall, the median score of 3 (IQR: 2.05) reflects neutrality, highlighting areas for improvement.

Analyzing instructors’ perceptions provides insights into the CEP’s effectiveness and areas for improvement. Data from 61 participants showed variability in responses across documentation, clarity, usability, and alignment with educational outcomes.

Only 30% agreed that the CEP effectively tracked student progress, while 34% disagreed. Similarly, 44% were dissatisfied with how it documents course objectives, highlighting gaps in aligning evaluation with educational goals. Concerns about student safety were notable, with 28% strongly disagreeing that the CEP fulfills this role.

Clarity of instructions and criteria also drew criticism. While 26% found the criteria clear, 44% disagreed, suggesting ambiguity. Similarly, 30% found CEP instructions unclear, indicating a need for better guidance.

Usability issues were evident, with 21% disagreeing that the tools are user-friendly and 39% finding the time requirements excessive. Alignment with expectations and orientation effectiveness were also questioned, with 33 and 44% expressing dissatisfaction, respectively.

The median score of 2.76 (IQR = 2.09) reflects a neutral to slightly negative perception, emphasizing the need for improvements in documentation, clarity, usability, and instructor preparation.

### Variations in students’ perceptions of CEPs and demographic characteristics

The analysis of students’ perceptions of CEPs highlights the influence of demographic factors (Table 3). Age had a significant impact, with students aged 24–26 reporting the highest perceptions (*M* = 3.48) and those aged 27–29 reporting the lowest (*M* = 2.67).

**Table 3:** Instructors’ perceptions by demographic variables.

Variable	Category	*N*	Mean	SD	*p*-value
Gender	Male	28	2.79	0.61	0.002
	Female	33	2.93	0.58	
Marital status	Single	13	2.64	0.62	0.009
	Married	48	2.84	0.55	
Teaching level	Level 5–6	19	2.81	0.60	0.515
	Level 7–8	42	2.87	0.57	
University	College A	35	2.86	0.56	0.17
	College B	26	2.82	0.59	

Subscale 1 (effectiveness and alignment) showed significant differences (*H* = 13.5, *p* = 0.009), while subscale 2 (clarity and usability) showed no variation (*H* = 6.0, *p* = 0.197). These findings suggest that age influences perceptions of CEP effectiveness more than clarity or usability.

Students’ levels of study did not significantly impact their overall perceptions (*H* = 2.52, *p* = 0.472). However, subscale 2 revealed notable differences across levels (*H* = 9.87, *p* = 0.019), with higher levels showing more favorable perceptions of clarity and usability. This finding suggests that students’ understanding of CEP tools improves as they progress through their studies.

Marital status of students significantly influenced perceptions in subscale 1, with single students scoring higher than married counterparts (*U* = 4,561, *p* < 0.001). However, no significant differences were found in the overall score of subscale 2.

Gender and university affiliation did not influence students’ perceptions, with no significant differences between total scores or subscales.

Age and study level were key factors, particularly in CEP effectiveness and clarity, while gender and university affiliation played minimal roles. Addressing these gaps can enhance program effectiveness and usability.

### Variations in instructors’ perceptions of CEPs based on demographic characteristics

The analysis of instructors’ perceptions of CEPs based on their demographic characteristics (Table 4) found no significant differences based on age, instructional level, or university affiliation. Perception scores across all age groups were consistent (*M* = 2.43–3.65, *H* = 1.24, *p* = 0.37), with subscales 1 and 2 showing similar uniformity. Teaching levels (levels 5–8) also showed no substantial variations (*H* = 1.19, *p* = 0.515), with total scores ranging from *M* = 2.73–3.25, suggesting stable perceptions across instructional levels.

**Table 4:** Clinical instructors’ perceptions of CEPs by demographic characteristics (expanded).

Variable	Category	*N*	Mean (SD)	Test	Statistic	*p*-value	Effect size
Gender	Male	28	2.79 (0.61)	Mann–Whitney U	440	0.002	*r* = 0.35
	Female	33	2.93 (0.58)				
Marital status	Single	13	2.64 (0.62)	Mann–Whitney U	273	0.009	*r* = 0.28
	Married	48	2.84 (0.55)				
Teaching level	Levels 5–6	19	2.81 (0.60)	Mann–Whitney U	–	0.515	*r* = 0.06
	Levels 7–8	42	2.87 (0.57)				
University affiliation	College A	35	2.86 (0.56)	Mann–Whitney U	–	0.170	*r* = 0.08
	College B	26	2.82 (0.59)				

Effect sizes interpreted as small (*r* ≈ 0.10), moderate (*r* ≈ 0.30), large (*r* ≥ 0.50).

Gender-based analysis revealed significant differences. Female instructors had higher overall perception scores (*M* = 2.93) than male counterparts (*M* = 2.79, *U* = 4.40, *p* = 0.002). Female instructors rated effectiveness higher (*M* = 3.15, *U* = 4.33, *p* = 0.008), while males scored higher on clarity and usability (*M* = 3.37, *U* = 5.68, *p* = 0.02).

Marital status significantly influenced perceptions, with married instructors scoring higher (*M* = 2.84) than single instructors (*M* = 2.64, *U* = 2.73, *p* = 0.009). Subscale scores followed similar trends in favor of married instructors.

University affiliation had no significant impact (*U* = 7.44, *p* = 0.17), as instructors across institutions rated CEPs similarly in effectiveness, clarity, and usability.

A multiple linear regression analysis was performed to examine the independent association between demographic variables and overall CEP perception scores. Age and academic level emerged as significant predictors among students (*p* < 0.05), while gender and marital status were significant predictors among instructors. The regression models explained a modest proportion of variance, indicating that demographic factors exert a small but statistically significant influence on perceptions of CEP.

## Discussion

This study examined nursing students’ and clinical instructors’ perceptions of Clinical Evaluation Programs within Saudi nursing education. Overall, CEPs were perceived as necessary but moderately effective, suggesting that, while their presence is valued, their implementation requires refinement. These findings align with previous studies, indicating that the success of clinical evaluation depends more on clarity, consistency, and feedback quality rather than the availability of tools.

### Nursing students’ perceptions of clinical evaluation programs

The findings of our study revealed that nursing students generally held a neutral perception of CEPs within their clinical settings. Students acknowledged the contribution of CEPs in documenting their clinical performance and promoting a safe working environment; however, concerns were raised regarding assessment criteria, evaluation instruments, and the timeliness of feedback. Similar perceptions were reported in studies conducted by Krautscheid et al. (16) and Alshammari et al. (17).

Importantly, these perceptions appear to be influenced not only by the evaluation tools themselves but also by instructor involvement and the clinical learning environment. Levett-Jones et al. suggested that limited instructor availability and inconsistent supervision may reduce students’ satisfaction with the assessment process (18). Pearson’s study supports this rationale, indicating that inconsistent instructor involvement can undermine students’ confidence in the credibility of the CEP assessment process. Some authors have suggested that inadequate feedback may demotivate individuals from performing well; however, this assumption was not supported by the participants in the present study (19, 20).

Notably, other studies have reported more positive student attitudes toward CEPs, particularly when highly structured simulation-based evaluations are implemented (21). The comparison can be attributed to varying levels of resource availability, faculty training, and the standardization of tools used for evaluations across institutions. This suggests that the quality of assessments may vary, depending on these factors rather than just their design.

### Influence of age and academic level on student perceptions

Significant associations were identified between age and perceptions related to the effectiveness of CEPs and their alignment with learning objectives. Older students reported more positive perceptions, which may be explained by greater clinical exposure, improved coping strategies for stressful situations, and a more established professional identity. Benner’s novice-to-expert model (2011) provides a useful framework for interpreting this finding, as more experienced students may have a better ability to contextualize assessment processes (22).

However, these findings contradict those of Al Hadid et al., who reported little difference in perceptions of clinical evaluation between different age groups. This discrepancy can be attributed to cultural differences and variations in curricula, with the Saudi nursing education system placing the responsibility for evaluating CEPs on senior students in their later years (23).

Similarly, students with a higher academic level showed a more positive perspective in regard to clarity and user-friendliness of evaluation processes, indicating that awareness of these processes facilitates a more favorable response. This confirms the findings proposed by Anisi et al. in 2025, indicating that anxiety associated with evaluation processes decreases with proper orientation and exposure (24). Students in the initial stages may feel perplexed or anxious due to unclear expectations.

### Influence of marital status on students’ perceptions

There were significant variations between married and unmarried groups related to perceptions on the effectiveness of CEPs, as well as time management, as married individuals tend to perceive these areas more positively. This can be attributed to a deeper value placed on an organized and expected evaluation process among students who must balance their academic responsibilities with family life. According to Gamage et al., “personal circumstances can shape the experience” of academic demands (25).

However, some studies have indicated a higher level of stress among married nursing students with competing responsibilities compared to the non-student group (26, 27). This result appears to be counterintuitive to the findings of this study. It may indicate the effectiveness of well-structured CEPs in reducing stress among students with extracurricular responsibilities.

### Clinical instructors’ perceptions of clinical evaluation programs

Clinical instructors’ perceptions of CEPs ranged from neutral to very positive. They recognized the significance of CEP but identified limitations regarding documentation efficiency, clarity, and the timeframe available for implementation. Their perceptions also support similar findings by Omer and Aslam, as well as Kanwal et al., which stated that overloaded teaching duties or respective preparation can lead to dissatisfaction with evaluation processes (28, 29).

The impression of subjectivity and inconsistency in evaluation turned out to be another important consideration. Though these instruments aim to improve objectivity, they have to be applied on a consistent basis. Interestingly, studies carried out by Marasi et al. showed that there was a great degree of satisfaction among instructors regarding CEPs when there were extensive faculty development programs implemented within the context (30).

### Gender and marital status differences among instructors

Female lecturers were more satisfied with the overall results achieved by the CEPs, while male lecturers were satisfied with their usability. The findings from previous studies conducted by Yen et al. in 2024 indicate that female lecturers are likely to focus on their relationship-development role as lecturers, as opposed to results-oriented strategies used in formative evaluations practiced in CEPs (31). On the other hand, male lecturers were more likely to focus on efficiency, which affected their levels of satisfaction with tools such as CEPs.

Married faculty members also tended to have more positive views, possibly because they experience less job instability. Comparable outcomes were reported by Alsubaie et al., who indicated that internal stability is crucial for job satisfaction and commitment to teaching duties (32). Despite this perspective, other research has found that marital status does not affect faculty views (33), as specific settings may influence this association.

### Implications and recommendations

Based on the findings above, CEPs could be supported in a variety of ways, including the standardization of evaluation methods, clear instruction for students and faculty, feedback systems, faculty development programs to think in terms of clinical evaluation strategies, early student orientation on CEP faculty expectations, and the inclusion of simulation-based assessments.

From a policy point of view, a reduced administrative burden placed on clinical instructors and an equal ratio of instructors to participants could enhance engagement with CEPs. Instead of adopting innovative technologies randomly, focus should be placed on optimizing CEP infrastructures through training, improving learning outcomes, and monitoring quality.

### Strengths and limitations

The inclusion of both nursing students and CEP facilitators is the strength of this study, as it provides a comprehensive perspective. Additionally, the use of measurement scales with high internal consistency enhances the reliability of the findings.

However, several limitations must be acknowledged. The use of convenience sampling limits the generalizability of the results and introduces the potential for selection bias. Furthermore, the cross-sectional design restricts the ability to infer causal relationships. Although effect sizes indicated small-to-moderate practical significance, other contextual factors—such as institutional characteristics and cultural influences—may also shape perceptions and warrant further investigation in future studies.

## Conclusion

In conclusion, this study demonstrates that nursing students and clinical instructors perceive Clinical Evaluation Programs as essential yet imperfect elements of clinical education. Perceptions are influenced more by clarity, feedback quality, and practical implementation than by demographic characteristics. Improving organizational aspects of CEPs, strengthening instructor training, and aligning the clinical assessment process with the needs of participants represent key steps to improving clinical education quality. Current findings represent evidence-based support for nursing professionals eager to optimize CEP effectiveness within the context of Saudi Vision 2030.

## Data Availability

The raw data supporting the conclusions of this article will be made available by the authors, without undue reservation.
